# Achalasia in an Eight-Week-Old Infant Successfully Managed by CRE Balloon Dilatation: A Case Report and Literature Review

**DOI:** 10.7759/cureus.69537

**Published:** 2024-09-16

**Authors:** Ali Alsarhan, Moataz Hamdi, Karen Golightly, Christos Tzivinikos

**Affiliations:** 1 Pediatric Gastroenterology, Al Jalila Children's Specialty Hospital, Dubai, ARE; 2 Hospital Medicine, Al Jalila Children's Specialty Hospital, Dubai, ARE; 3 Rehabilitation Medicine, Al Jalila Children's Specialty Hospital, Dubai, ARE

**Keywords:** achalasia, balloon dilatation, esophagus, pediatric, therapeutic endoscopy

## Abstract

Achalasia is uncommon in pediatrics and typically presents after five years of age. It is often managed medically, endoscopically, or surgically such as myectomy. This case highlights an exceptionally rare occurrence of achalasia at the age of eight weeks, successfully treated with endoscopic CRE® balloon dilatation, providing prompt relief of symptoms. From birth, this full-term infant experienced persistent vomiting and choking, coupled with suboptimal weight gain and unresponsiveness to anti-reflux measures. Diagnostic assessments revealed notable findings a barium meal demonstrated contrast pooling in the esophagus with distal rat tail narrowing, and esophageal manometry identified elevated pressure at the lower esophageal sphincter (LES) and inadequate esophageal contractions consistent with the diagnosis of achalasia. The infant underwent two sessions of esophageal CRE® balloon dilatation under fluoroscopy 10 days apart. Possible associated syndromes were ruled out. No further interventions were needed for a follow-up duration of one year. The literature review reveals several modalities for treating achalasia in adults and older children, but there is a scarcity of data on younger children and infants. In this article, we reviewed current available evidence regarding treatment modalities and the success rate. There is an obvious lack of recommendations for children, particularly at younger ages, and the outcome is various given the rarity of this condition and the limited experience.

## Introduction

Effective esophageal motility and function are essential for feeding in children, playing an important role in propelling food particles down the esophagus during feeding. Various disorders can affect the esophagus, including rare disorders, and achalasia is among them. Achalasia is a neurogenic dysmotility disease that occurs in about 1.1 cases in a million children [1]. Oftentimes, less than 5% of symptomatic cases of achalasia present in patients younger than 15 years old [2]. It tends to happen more in males [1]. Statistically, disease rates vary among different countries [3]. This could hypothetically be attributed to variations in reporting levels or differences in environmental and genetic factors. Achalasia is an autoimmune-related disease with the destruction of neurological cells of the esophageal wall, but the exact cause is unclear [1]. Here, we report a rare case of achalasia in a very young infant. We propose that the condition was established since birth, but it came to medical attention and diagnosis was made at eight weeks of age.

## Case presentation

An eight-week-old male infant, delivered at 37 weeks of gestation and weighing 3 kg at birth, presented with symptoms of feeding difficulties, including choking, vomiting, poor oral intake, and poor weight gain since birth. Recent exacerbation of symptoms, characterized by postprandial vomiting, led to hospital admission. Although the infant appeared well-nourished and hydrated, his growth has been suboptimal. Despite the administration of an anti-reflux formula, the infant continued to exhibit feeding challenges, with audible gurgling sounds during feeds. A comprehensive evaluation was done at another hospital a few days earlier, including an echocardiogram, abdominal ultrasound, brain ultrasound, and newborn screening. All tests reported normal results. His birth history was significant for choking on milk and turning blue, necessitating NICU admission. Initial concerns pointed to tracheoesophageal atresia with fistula, but a barium swallow indicated slow milk passage through the esophagus.

Blood tests were repeated in our hospital, and he had a low hemoglobin level of 6.3 g/dL, with normal white blood cell and platelet counts. Reticulocytes were elevated at 3%. Hemoglobin electrophoresis and iron studies were normal. A blood film analysis showed irregularly shaped red blood cells with microcytic hypochromic cells, ovalocytes, and anisopoikilocytosis. Liver function tests, renal function tests, and electrolytes were within normal ranges.

**Figure 1:**
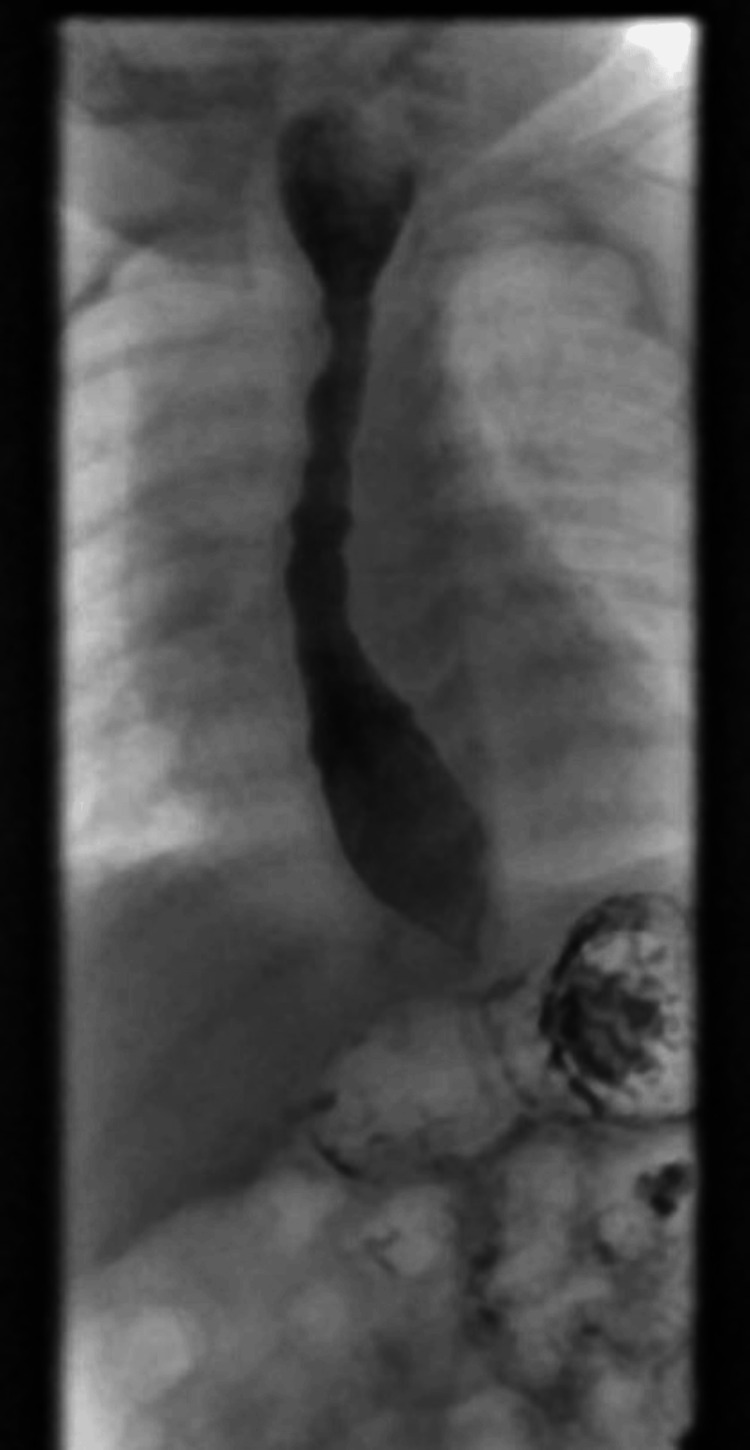
Barium esophagogram showing narrowing of the distal esophagus with proximal dilatation (ballooning) with secondary contractions.

**Figure 2:**
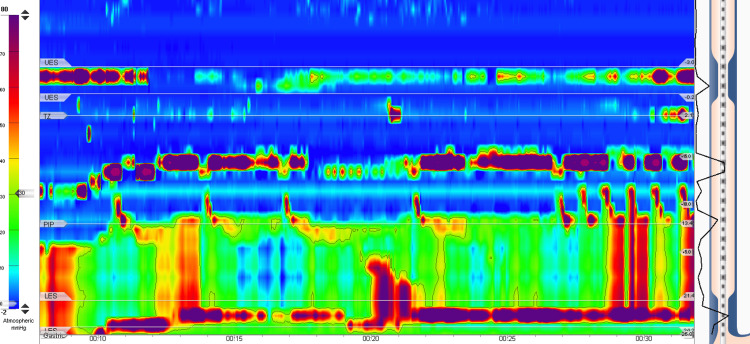
Esophageal manometry showing persistent elevation in the pressure of the lower esophageal sphincter.

Following an evaluation by a speech and language pathologist, which confirmed normal swallowing, the patient underwent an upper gastrointestinal barium study. The assessment revealed normal oral coordination, but the images indicated progressive dilatation and widening of the esophagus, and a symmetric smooth narrowing resembling a beak at the gastroesophageal junction (Figure 1). In addition, there was observed cranial and caudal propulsion of the contrast material without complete clearance. Delayed images showed the persistent presence of barium contrast in the esophagus. Subsequently, esophageal manometry (limited study) was performed, revealing a non-relaxed high-pressure distal esophageal sphincter (Figure 2).

On the following day, the patient underwent endoscopic esophageal balloon dilatation in the operating room. The mucosa of the esophagus appeared normal, but the distal part was narrowed without stricture or mass. A CRE® balloon catheter was inserted alongside the scope under fluoroscopy guidance, positioned at the gastroesophageal junction. The balloon was inflated sequentially to 6, 7, and 8 mm for one minute each. No mucosal tears or bleeding were observed. A contrast study was conducted, revealing no contrast leak at the gastroesophageal junction, which was located 18 cm from the gingiva. The contrast freely passed into the stomach. A nasogastric (NG) tube was inserted and secured at 23.5 cm, but the patient removed it upon waking up. Subsequently, the patient resumed normal feeding, and all symptoms were resolved. However, after 10 days, the patient returned with the same initial complaint, and he was admitted for another successful esophageal dilatation using the CRE® balloon catheter, but this time inflating to 8, 9, and 10 mm for one minute each, without complications.

It is worth mentioning that he underwent whole genome sequencing, which did not reveal any known gene mutation. In addition, his cortisol level was normal, and his esophageal histopathological analysis did not show any abnormality. Collectively, the associated syndrome was ruled out. At a one-year follow-up, he gained weight appropriately and did not need any further intervention. Barium esophagogram showed narrowing but contrast flew freely with no delay.

## Discussion

Achalasia is a disease that results from the degeneration of the neurons in the lower esophageal sphincter (LES). This process is selective, which damages the inhibitory cells in the myenteric plexus [4]. In a genetically susceptible individual, possibly after an environmental trigger whether a virus or something else, the immune system reacts and causes the damage [1]. CD3/CD8-positive cytotoxic T cells in addition to mast cells and eosinophils have been found in the tissue samples obtained by autopsy [1]. Auto-antibodies to myenteric neurons have been found as well [5]. Moreover, there is a reduction of nitric oxide (NO), interstitial cells of Cajal (ICCs), and vasoactive intestinal peptide (VIP), which are neurotransmitters responsible for inhibitory signals and muscle relaxation [4]. In animal models, alteration in the NO synthase 1 gene was associated with a higher LES tone and poor relaxation [5]. On the other hand, viral infections, particularly herpes simplex virus (HSV), are proposed and investigated as a potential factor to induce the immune system leading to the destruction of the neurons in the esophagus [5].

Achalasia is rare in children compared to adults, with a lower incidence observed in younger age groups. In the United Kingdom (UK), a study reported 228 cases of achalasia from 24 medical centers, and only 9% were children under four years old [6]. In the Netherlands, a retrospective study found that only 14 % of the cases were under the age of eight years [7]. There is a limited number of cases reported in infancy, and a majority of them are in late infancy [8]. There are some reports of infants with achalasia who were identified to have syndromes such as Allgrove syndrome (or triple A syndrome) or Achalasia-microcephaly syndrome [9]. The rarity, small size, and low weight, coupled with associated medical problems, pose challenges in managing and tailoring treatment for individuals with this condition during infancy.

Diagnosis is usually delayed for a few years because of the resemblance of symptoms in achalasia with other disorders such as gastroesophageal reflux (GERD) [1]. Symptoms can range from vomiting to dysphagia, regurgitation, and weight loss [1]. The individual might be treated using different medications without resolution of symptoms until the diagnosis of achalasia is established, a process that can take up to 10 years from the onset of complaints [1]. An esophagogram is utilized to demonstrate the dilatation of the esophagus with tapering of the distal LES into what is typically known as a “bird's beak” [1]. This modality can detect up to 60% of cases while 40% can be missed [10]. The enhanced technique is the “timed barium swallow," which assesses barium retention and column height in the esophagus at one, two, and five minutes [11]. In adults, the suggested cutoff is 5 cm of the barium column at two minutes, providing a sensitivity and specificity of approximately 85% for diagnosing achalasia [11]. The cutoff in children is not established.

While upper endoscopy is crucial for treatment, it plays a pivotal role in assessing patients and excluding other causes of LES narrowing, such as tumors or strictures. In addition, it allows the obtaining of biopsies to rule out mucosal pathologies such as severe GERD or eosinophilic esophagitis [10]. Esophageal mucosal biopsies can show thickening of the basal layer, widening of the intercellular spaces, enlarged squamous cells with intracellular edema, and increases in intraepithelial lymphocytes [12]. A muscular biopsy, typically not obtained during regular endoscopic procedures, can demonstrate the absence of ganglion cells in up to 88% of cases [12]. Furthermore, muscular atrophy and interstitial fibrosis may also be present [12].

The gold-standard diagnostic modality is high-resolution esophageal manometry (HRM) [10]. Chicago classification (CC) is used to evaluate esophageal motility disorder [10]. There are three subtypes of achalasia based on CC [10]: type I, with abnormal median integrated relaxation pressure (IRP), no contractility (no peristalsis); type II, with abnormal median IRP, no contractility (no peristalsis), with >20% swallows showing pan-esophageal pressurization; and type III, with abnormal median IRP, >20% swallows with premature or spastic contractions, and no peristalsis.

Different types of achalasia yield varying outcomes after therapeutic intervention. Type II has the best outcome (96%), type I is intermediate (81%), and type III is the least favorable (66%) [13].

The utilization of HRM is limited by age. Children under four years face challenges in following instructions and the protocol required for HRM procedures [14]. In the pediatric age group, including term and pre-term babies, the fifth to 95th percentiles are used to determine the normal ranges for the resulting parameters [14]. We had difficulty performing the procedure on our patient. The catheter was large and difficult for insertion in his small nostrils, which caused him significant discomfort. In addition, it was difficult to keep the child calm and follow the Chicago protocol strictly. Eventually, we were able to demonstrate elevated pressure in the LES and the lack of peristaltic movement in the esophagus.

It is essential to record any medication used during the procedure and within 24-48 hours earlier [14]. Prokinetics and anticholinergic agents should be withheld for two days [14]. On the contrary, midazolam or ketamine can be used during the procedure without affecting esophageal and LES muscular tone [14]. Opiates should be avoided due to their impact on esophageal motility [14].

The patient described in this report underwent balloon dilatation. Initially, a cautious dilatation of up to 8 mm was performed, resulting in symptomatic resolution. After 10 days, another dilatation was necessary, this time reaching 10 mm. The precise number of sessions required for the child is uncertain, but it is likely that surgical intervention may eventually be needed. The dilation is recommended to be done using a pneumatic balloon (PB), which can be inflated to 30 to 40 mm in order to split the circular muscle and loosen the LES [13]. Due to the small size of the baby, we were unable to use the PB, and alternatively, the CRE balloon was used. Pastor et al. found that 17% of children who underwent PB dilation did not necessitate additional intervention, whereas 53% required further dilatation episodes without any need for myotomy [1]. His findings provided optimism regarding the potential resolution of the problem without the need for surgery. Overall, PB dilatation success rate in children is 60-80 % [15]. The European Achalasia Trial investigators found that pneumatic dilation or laparoscopic Heller myotomy (HM) in adults was equally effective at a two-year follow-up [1]. However, HM would be preferable in patients younger than 40 years of age, which can promise a longer duration of symptom resolution [1]. A wider diameter of PB was associated with a higher rate of success but a greater risk of complications such as perforation, intramural hematoma, or GERD [5,7]. 

HM is performed laparoscopically, and there has been a modification involving an incision solely in the anterior wall of the esophagus, whereas it previously included the posterior wall as well [1]. In the pediatric age group, several reports have demonstrated a higher rate of success and safety of laparoscopic HM over PM dilatation [1]. Contrary to that, a systematic review has failed to show the superiority of laparoscopic HM over PM dilatation [1]. As per Pacilli and Davenport, laparoscopic HM has an overall success rate of 85%, and it has been performed in patients younger than one year of age [15]. A case report was published in 2018 of a 45-day-old infant who weighs 3.2 kg and has undergone HM procedure successfully [16]. The procedure can be combined with fundoplication; however, whether the HM was performed in conjunction with fundoplication or not, it did not change the likelihood of developing GERD after the procedure [15].

Alternative treatment methods, including medications (such as calcium channel blockers, nitrates, and sildenafil) or interventional techniques (like botulinum injection and peroral endoscopic myotomy (POEM)), exist; however, each comes with its own set of limitations [1]. Medications are partially effective and do not address the underlying defect. Botulinum injection provides transient relief, but symptoms tend to recur after a few months. POEM is technically challenging and can only be performed in a limited number of specialized centers globally [1]. Ultimately, reaching a conclusion regarding the most suitable interventional modality remains a controversy. However, in practice, we often opt for esophageal dilatation and Heller myotomy for patients who are suitable for these procedures.

## Conclusions

We report a case of achalasia in an eight-week-old male, which is a rare neurogenic dysmotility disorder, particularly in infants, and poses significant therapeutic challenges. Balloon dilatation provides a temporary relief but was a permanent solution in this case as we have demonstrated. Our literature review could not identify any particular intervention that is recommended in this age group. Ongoing research and case studies are needed to enhance our understanding and management of achalasia in this vulnerable population.
